# Highlighting an atypical cause of the Face of the Giant Panda sign

**DOI:** 10.1259/bjrcr.20170046

**Published:** 2017-08-04

**Authors:** Stephanie Vella, Reuben Grech

**Affiliations:** ^1^Department of Medicine, Mater Dei Hospital, Msida, Malta; ^2^Department of Medical Imaging, Mater Dei Hospital, Msida, Malta

## Abstract

The “Face of the Giant Panda” and the “Panda Cub” signs are neuroimaging features originally described in patients with Wilson’s disease. We present a case with similar imaging findings in a different clinical context and highlight other differential diagnoses to be considered when presented with this particular radiological sign.

## Cinical presentation and imaging findings

**Figure 1.**
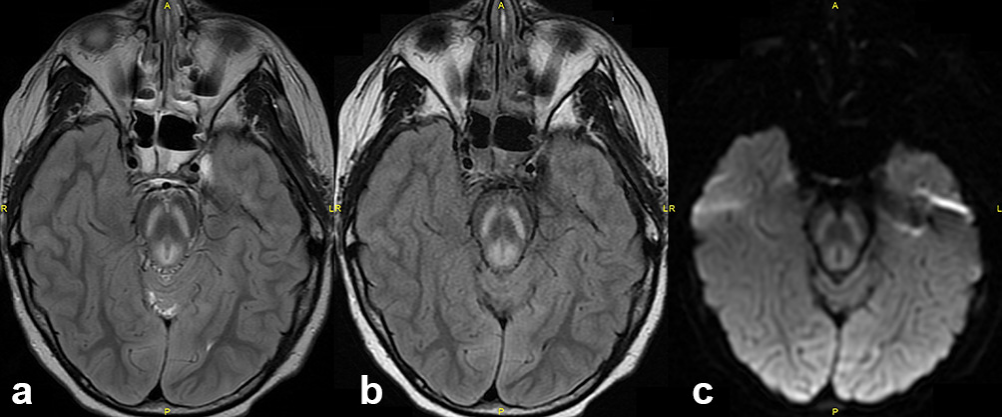
Axial *T*_2_ weighted (a) and FLAIR (b) MRI images of a 24-year-old female who suffered hypoxic brain injury. These demonstrate symmetrical high signal intensity in the mid-brain, thalami and hippocampi. In the mid-brain, the abnormal signal intensity affects the substantia nigra and the periaqueductal grey matter with sparing of the red nuclei and cortical spinal tracts. Diffusion weighted imaging (c) demonstrates increased high signal intensity corresponding to affected regions. FLAIR, fluid attenuation inversion recovery.

**Figure 2.**
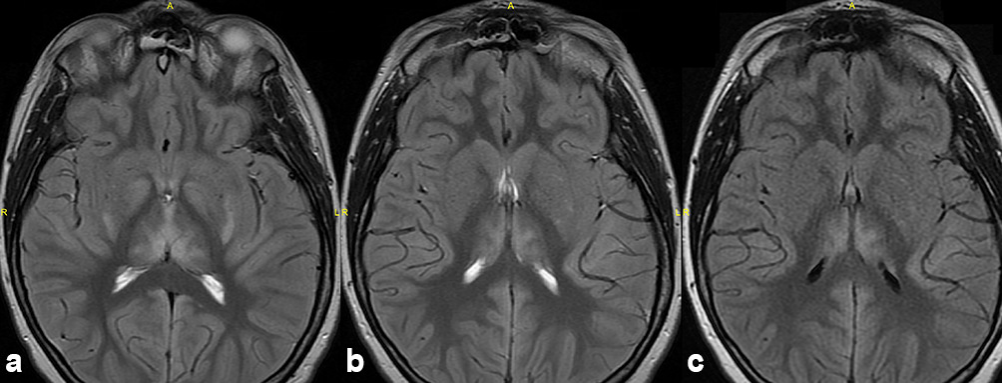
MRI brain images of the same patient in Figure 1. Axial *T*_2_ weighted images (a, b) demonstrate symmetrical hyperintensity in the thalami and basal ganglia regions. Axial FLAIR (c) MRI image shows no hyperintensity involving the internal capsule, and the head of the caudate nucleus is not atrophied. FLAIR, fluid attenuation inversion recovery.

A 24-year-old female on holiday in Malta was found unresponsive at her residence. Cardiopulmonary resuscitation was commenced in view of cardiac arrest *en route* to hospital. According to her partner she was a known case of systemic lupus erythematosus with no other comorbidities. Return of spontaneous circulation was obtained after prolonged cardiopulmorary resuscitation; however, the patient remained comatose and intubated in intensive care. MRI of the brain demonstrated pathologically symmetrical high signal intensity in the mid-brain, thalami, basal ganglia and hippocampi. In the mid-brain, the abnormal signal intensity affected the substantia nigra and the periaqueductal grey matter with sparing of the red nuclei and cortical spinal tracts (Figures 1 and 2). This resulted in an appearance similar to the “Face of the Giant Panda” sign. In our case the findings were secondary to hypoxic-ischaemic encephalopathy with no past history to suggest hepatolenticular degeneration. The patient unfortunately passed away after a few days from multiorgan failure.

## Discussion

The “Face of the Giant Panda” sign was first described in 1991 by Hitoshi *et al*.^1^ in patients with Wilson’s disease, an inherited inborn error of copper metabolism caused by a mutation of the copper-transporting gene *ATP7B* resulting in copper deposition the basal ganglia, liver and cornea among other organs.

*T*_2_ weighted brain MRI in patients with Wilson’s disease is usually notable for hyperintensity of the putamen, globus pallidus, internal capsule and thalamus. Additionally atrophy of the head of caudate nucleus, brainstem and cerebral and cerebellar hemispheres may also be evident. The “Face of the Giant Panda” is a result of high signal intensity of the tegmentum with normal hypointense red nuclei forming the eyes, preserved signal intensity of the substantia nigra pars reticulata forming the ears and hypointensity of superior colliculi forming the chin. The pontine “Panda Cub” sign has also been described, in which the hypointense medial longitudinal fasciculus and central tegmental tract (eyes) and hyperintense aqueduct (nose and mouth) result in this typical appearance.^1–3^

MRI provides useful information with regards to metal deposition; however, the exact mechanism behind the appearance of the “Face of the Giant Panda” sign remains unknown. It is postulated that this may be due to the paramagnetic effects of copper and iron.^4,5^ Regression in MRI changes has been reported following chelation therapy or orthotopic liver transplantation.

Differential diagnoses which may produce a similar MRI appearance include Leigh disease; a progressive neurodegenerative disorder leading to death in early childhood, Japanese B encephalitis; a vaccine preventable disease caused by the mosquito-borne Japanese encephalitis virus, methanol toxicity; where formic acid results in optic nerve damage and encephalopathy, and extrapontine myelinolysis; seen in alcohol abusers, malnourished patients and in rapid correction of hyponatremia.^6^

## Learning points

1. The “Face of the Giant Panda” and the “Panda Cub” signs are classically described neuroimaging appearances seen in patients with Wilson’s disease.
2. A number of conditions may demonstrate the “Face of the Giant Panda” sign and should be considered depending on the clinical scenario.
